# Papillomaviruses and Endocytic Trafficking

**DOI:** 10.3390/ijms19092619

**Published:** 2018-09-04

**Authors:** Abida Siddiqa, Justyna Broniarczyk, Lawrence Banks

**Affiliations:** 1 Tumour Virology Laboratory, International Centre for Genetic Engineering and Biotechnology, I-34149 Trieste, Italy; Abida.Siddiqa@icgeb.org (A.S.); justekbr@gmail.com (J.B.); 2 Department of Molecular Virology, Adam Mickiewicz University, Poznan 61–614, Poland

**Keywords:** HPV, viral capsid proteins, viral oncoproteins, endocytic machinery, retromer, retriever, sorting nexins

## Abstract

Endocytic trafficking plays a major role in transport of incoming human papillomavirus (HPVs) from plasma membrane to the trans Golgi network (TGN) and ultimately into the nucleus. During this infectious entry, several cellular sorting factors are recruited by the viral capsid protein L2, which plays a critical role in ensuring successful transport of the L2/viral DNA complex to the nucleus. Later in the infection cycle, two viral oncoproteins, E5 and E6, have also been shown to modulate different aspects of endocytic transport pathways. In this review, we highlight how HPV makes use of and perturbs normal endocytic transport pathways, firstly to achieve infectious virus entry, secondly to produce productive infection and the completion of the viral life cycle and, finally, on rare occasions, to bring about the development of malignancy.

## 1. Introduction

High-risk human papillomaviruses are the main etiological agent of many human malignancies, with cervical cancer being the most common. Over 200 HPV types have been reported. HPVs have an 8 Kb double-stranded circular genome that encodes early and late proteins. The early ORFs encode E1, E2, E5, E6 and E7, whereas the late ORFs encode E4, L1 and L2. The viruses replicate in differentiating epithelia, and gain access to the basal cell through small lesions in the skin. Once infected, the basal keratinocyte begins to differentiate and the viral oncoproteins, E6 and E7, block tumor suppressor activity and promote cell cycle entry and replication of the viral genomes. This results in the production of new infectious virus particles in the upper terminally differentiated layers of the skin [1]. In rare cases, this infectious cycle is perturbed, which can ultimately result in the development of cancer [2].

The late proteins L1 and L2 play an essential role in establishing viral infection and later they encapsidate the newly-synthesized viral genome. L1 facilitates binding of the virus to the extracellular matrix (ECM) through laminin-332 [3], and to cell surface receptors, including heparan sulfate proteoglycans (HSPGs) [4,5,6,7]. This binding causes conformational changes in the capsid, facilitated by cyclophilin B, which results in exposure of the L2 N-terminus [8,9]. In addition, secreted trypsin-like serine protease kallikrein-8 (KLK8) has been shown to cleave the L1 capsid, independently of cyclophilin B, and this also causes a structural change in the capsid to promote infection [10].

One of the most critical steps in the infectious entry of PV is the furin cleavage of this exposed L2 N-terminus for endosome escape [11,12,13], although pre-cleavage by furin has been suggested to occur for some HPV types during virus morphogenesis, obviating the requirement for this post cell binding [14]. The virus then is up taken by an as yet unknown secondary receptor, which also appears to require signaling and active proliferation of the target cell [15,16,17,18]. During intracellular trafficking, the acidification of endosomes is an important event that results in virus uncoating, after which most of the L1 protein segregates from the L2/viral DNA complex into an endocytic compartment and is targeted for degradation at lysosomes. The L2 and viral DNA (L2/vDNA), together with a small amount of L1, egress from the endosome and accumulate in the TGN [19]. Cell cycle progression is considered important for HPV infection, and nuclear envelope breakdown is required for the L2/vDNA to transfer from the TGN into the nucleus [20,21,22]. The L2/vDNA complex eventually resides at promyelocytic leukemia (PML) nuclear bodies, which are thought to support the early viral gene expression that is required for the establishment of infection [23].

Despite our understanding of the role of HPV in cellular transformation and cancer progression, the early events in infection are still not completely understood. In this review, we summarize advances in the study of endocytic transport, with a focus on trafficking routes used for PV infection, the cellular sorting machinery and its role in establishing infection, as well as examining how HPV oncoprotein modulation of transport pathways can lead to cancer development.

## 2. Virus Entry

Endocytosis is a key process by which cells internalize macromolecules, pathogens and surface proteins. It plays an important role in cell-to-cell communication, as well as in intracellular trafficking. There are a number of different pathways involved in endocytosis, including clathrin-mediated endocytosis (CME) [24], caveolae uptake [25], cholesterol-sensitive clathrin and caveolae-independent pathways [26,27], clathrin independent carriers (CLIC) and glycophosphatidylinositol-anchored protein-enriched endosomal compartments (GEEC)—termed the CLIC/GEEC pathway [28]. No generalized mechanism for the mode of papillomavirus endocytosis has been observed: it varies depending on cell type and virus. For instance, some have used specific inhibitors to show that HPV-16, HPV-31 and BPV-1 exhibit clathrin-mediated endocytosis (CME) [29,30,31], while others indicate that HPV-16 and HPV-31 use dynamin-2 and caveolar/lipid-raft mediated endocytosis [29,32,33] respectively. However more recently, siRNA mediated knockdown of specific components of endocytosis shows that HPV-16, HPV-31 and HPV-18 entry is clathrin, caveolin, lipid raft and dynamin-independent and rather depends on actin, or occurs through tetraspanin-enriched microdomains (TEM) [34,35,36]. CD151 tetraspanin and its associated intergrin partner α6β1, annexin A2 heterotetramer (A2t), and the cytoskeletal adaptor obscurin-like 1 (OBSL1) have all been associated with TEM-dependent HPV-16 entry in different studies [35,37,38,39,40,41].

## 3. The Early Endosome

Endocytosed cargoes are sorted in the early endosomes (EE) to different destinations—back to the plasma membrane (recycling), or to lysosomes for degradation [42]. Several groups have shown localization of HPV16 and HPV31 with early endosome antigen 1 (EEA1)-positive compartments in a Rab5 GTPase-dependent manner [43,44,45]. This is an early event which has been reported as early as 5 min post-infection and can extend out to 4 h post-infection [43,44].

## 4. Endosomal Maturation and Lysosomal Degradation

The early endosome matures into the late endosome (LE), when it fuses with lysosomes and most of the content is degraded by lysosomal degradation [46]. One of the important aspects of the LE is the formation of the multivesicular bodies (MVBs) that contain intralumenal vesicles (ILVs), which play a role in sorting cargoes either for degradation in the lysosomes or for recycling [46,47]. These ILVs are formed with the help of the endosomal sorting complex required for transport machinery proteins (ESCRT-0, -I, -II, and -III), and the accessory proteins such as VPS4 and Alix. CD63-syntenin-1-ALIX complex is considered to play a role in the regulation of post-endocytosis trafficking of HPV-16, HPV-18 and HPV-31 to multivesicular endosomes, where capsid disassembly takes place, leading to uncoating and retrograde transport of the L2/vDNA complex to the TGN [48]. It was also shown that the loss of ESCRT machinery components TSG101 and VPS4 leads to a reduction in the infection levels of many different PVs, owing to a defect in capsid uncoating, showing that this is an evolutionarily conserved phenomenon in PV infectious entry [49,50]. Formation of ILVs was strongly reduced with depletion of TSG101 and VPS4 that results in accumulation of cargoes within the endosomes [51,52]. However, it is not known whether the loss of infection is due to TSG101 and VPS4-dependent endosomal perturbation [51,52], or whether it has a direct role in sorting of the L2/vDNA viral cargo. A recent study has also shown a role for A2t in virus progression from EE to MVBs, where depletion of A2t inhibits trafficking of virus to MVB owing to reduction in capsid uncoating and an apparent increase in lysosomal degradation of L1 [53].

In addition, sorting in the LE depends on endosomal acidification. Ligand–receptor complexes that are internalized in the endosome have different pH sensitivities, thus assisting the dissociation of receptor from ligand and allowing the recycling of cellular receptors [54]. For instance, cargoes (e.g., mannose-6-phosphate receptor) that are targeted to enter the TGN release their ligand in the LE where the pH is ~5.5 [54]. Endosomal acidification is considered an important step for PV as well [55], where CyPs mediates the separation of L2/vDNA complex from L1 during virus uncoating [56]. Most of the L1 is believed to be degraded, as indicated by the localization of L1 in LAMP1-positive vesicles [34,36,45,57]. However, several studies have shown that not all of the L1 protein dissociates from the L2 during uncoating [22,58,59,60] and later it was established that a small amount of conformationally intact L1 protein remains associated with the viral genome and L2 and accompanies the viral cargo to the TGN and nucleus [19].

## 5. Egress from the Late Endosome and Entry into the TGN

After egress from the LE, the incoming viral cargo enters the TGN [61], where only a portion of the L2 protein is accessible to the cytosol and the vDNA remains in a vesicular compartment during transport. This is a critical step in virus infection, as the block in egress from the LE leads to reduction/abrogation of infection. For instance, α-defensin HD5, a class of innate immune effectors that have an antiviral and antimicrobial effect against pathogens, can inhibit virus infection by altering L2/vDNA retrograde trafficking and redirecting it to the lysosome for degradation [4,62]. In the same manner, interferon gamma treatment results in a reduction of L1 degradation in lysosomes and retention of L2/vDNA in the LE, thus preventing the cargo from reaching the TGN [63]. Stannin is another small endosomal transmembrane protein that has been shown to reduce HPV-16, HPV-18, and HPV-5 virus infection without affecting virus uptake or virus uncoating, but instead blocking virus entry into the TGN by routing the cargo to lysosomal compartments for degradation. Stannin overexpression reduces L2 and VPS35 co-localization, suggesting that it abrogates L2-retromer binding, a critical step for L2/vDNA to enter the TGN [64]. Gamma (γ)-Secretase also happens to play a vital role in successful HPV entry. It is a multiprotein transmembrane protease complex, comprising four subunits: presenilin1/2 (PS1 and PS2), nicastrin (Nic), anterior pharynx defective 1 (APH-1), and presenilin enhancer 2 [65]. γ-Secretase is best known for cleaving the vast majority of type I transmembrane proteins, however type II transmembrane [66] and multipass transmembrane proteins (glutamate receptor subunit 3) also undergo PS-dependent cleavage [67]. Inhibition of γ-secretase or knockdown of one of the components of this protease results in significant reduction of HPV infection [68,69,70]. Sensitivity to this protease is conserved among alpha and beta HPV types [71]. Although an interaction between L2 and γ-secretase has not been reported, nor has the cleavage of L1 and L2 by γ-secretase been observed [68,70]. This suggests that γ-secretase might be cleaving an unknown cellular substrate(s) to allow infection to proceed. The exact role of γ-secretase in PV infection is unknown, but inhibition of γ-secretase activity blocks L2/vDNA from reaching the TGN, as mentioned before, although L2 seems to exit the early endosome, as indicated by staining of EEA1-positive compartments [69], which is in contrast to the effect on infection with a retromer block, where virus accumulates in the early endosome [72]. This suggests that γ-secretase is required in HPV entry after retromer action [69].

## 6. Endosomal Tubulation

**Figure 1:**
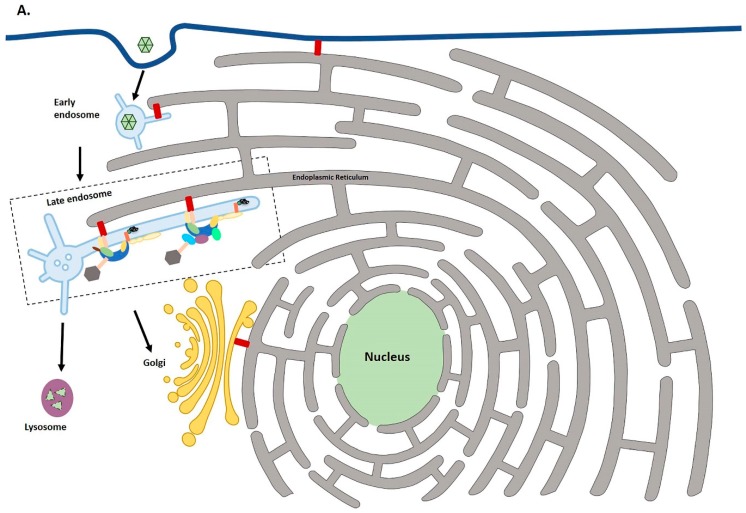

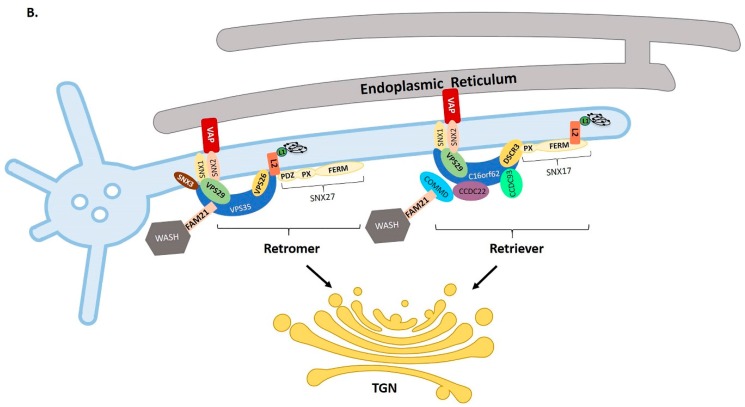
Schematic diagram of HPV intracellular trafficking to the TGN. (**A**) Following endocytosis, uncoating of papillomaviruses is triggered by acidification of the late endosome. Endosomes as they mature exhibit tubulation. L2/vDNA segregates from most of the L1, which is then degraded in the lysosome. L2/vDNA along with a small amount of L1 enters the TGN with the help of cellular sorting factors. The dashed box shows how the retromer and retriever complexes might cooperate in HPV infection, which is shown in more detail in panel B. (**B**) Prior to the entry of viral cargo to the TGN, L2 binds SNX27, retromer and SNX17 which is part of the retriever complex. The ER makes contact points with endosomes, Golgi and plasma membrane using integral ER VAP protein.

Early endosomes (EEs), as they mature, exhibit tubulation, which is an important factor in cargo sorting and recycling, along with endosomal acidification. EEs are spherical in shape, with a diameter of ~100–500 nm [46], which increases the ratio of the volume-to-surface area. This allows the accumulation of ligand-dissociated receptors into the lumen of vacuolar regions. On the other hand, the tubules that extend from the endosomes have more surface area-to-volume ratio, which facilitates the accumulation of transmembrane receptors and other cargoes for recycling or trafficking [46,73]. Besides endosomal acidification and geometry, efficient cargo sorting in the endosomes depends on the interaction of different protein complexes with the cargo molecules [74]. This association typically takes place along endosomal tubules. The mechanism of endosomal tubule formation is largely unclear, however it is known that it is driven by BAR (Bin, amphiphysin, Rvs) domain-containing proteins, which induce membrane curvature [75]. As mentioned above, these tubules provide a high surface area to volume ratio, which make them ideal for export of cargoes to their destinations [76,77]. The sorting factors that bind to the tubulating endosome for the transport of incoming papillomavirus are described in Section 8 and shown in Figure 1.

## 7. ER-Endosome Contact Points

There is an emerging concept in endosomal biology known as the ER-endosome contact site, first established when an association was found between the integral ER protein VAP-A and the peripheral late-endosomal cholesterol-binding protein ORP1L [78]. Before EE, LE or tubulating endosomes undergo fission, they form a contact point with the ER marked by retromer-associated protein FAM-21, which defines the position and timing of endosome fission [79]. Therefore, endosome maturation and trafficking is coupled to the ER contact points and the number of these contact points increases as endosomes mature [80].

Depletion of VAP-A and VAP-B results in accumulation of PI4P on endosomes, which leads to disruption of transport from endosomes to TGN, caused in part by dysfunction of the retromer and WASH complex [81]. It has been recently shown that HPV-16 induces endosomal tubulation, as determined by MICAL-L1 (molecules interacting with CasL-like 1) imaging. The endosomal tubulation depends on VAP proteins, without which the incoming virus fails to reach the TGN. It seems possible that VAP dependent ER-endosome contact ensures ER mediated cleavage of virion containing vesicles and hence facilitates the trafficking to the TGN. The increase in tubulation is an early event, as it can be observed at 2 h post-infection, reaches a maximum at 8 h post-infection and is greatly reduced by 24 h post-infection. However, it is not yet clear how incoming virions affect endocytic tubulation, nor how HPV uses this to its advantage [82].

## 8. L2 Membrane Spanning

When naturally expressed, L2 is a soluble nuclear protein, not a membrane protein [83,84]. However, during PV infection, a portion of L2 spans the endocytic membrane and allows interaction with a variety of cell-sorting factors in the cytosol to direct the cargo towards the TGN. These cytosolic cellular factors include, but are not limited to, the retromer complex, SNX17 and SNX27. Retromer is a well-known protein complex that plays an important role in retrograde transport of the cargoes. It consists of a cargo recognition complex (Vps26, Vps29, and Vps35), which is evolutionarily conserved in mammals, and a sub-complex of sorting nexin (SNX 1/2 or SNX 5/6) proteins that plays a role in membrane recruitment and in the formation of recycling tubules [85,86]. These SNXs are recruited to the endosome through the association of their Phox (PX; Phagocyte Oxidase) homology domain with PI3P in the endosomal membrane [87], whereas the cargo recognition complex is recruited by the interaction of VPS35 with Rab7 [88]. The SNX protein family has a SNX-FERM group, which includes SNX17, SNX27 and SNX31. The FERM domain plays a role in determining the destination of cargoes. SNX17 and SNX31 share roughly 40% amino acid sequence similarity and they only contain the SNX–PX and FERM-like domains, whereas SNX27 has an additional post-synaptic density 95/discs large/zonula occludens (PDZ) domain. This PDZ domain has an additional β-strand in the loop between strands β2 and β4, which gives it an arrangement of seven β-strands rather than the six β-strands of a typical core PDZ domain structure [89]. Typically, PDZ domains associate with other proteins through a PDZ-binding motif (PBM), which is a short peptide, usually at the C-terminus of the partner protein. SNX27 associates with the retromer complex and promotes recycling of cargoes from endosomes to the plasma membrane by linking PDZ-dependent cargo recognition to retromer-mediated transport. The SNX27 PDZ domain interacts directly with the VPS26 subunit of retromer, and this association is necessary and sufficient to prevent the lysosomal entry of SNX27-bound cargoes [90]. Interestingly, SNX27 has also been shown to interact directly with FAM21, a component of the WASH complex, which binds also retromer. This multiprotein SNX27-retromer-WASH complex (where FAM21 interacts with FERM and VPS35; and SNX27 PDZ interacts with VPS26) is required for recycling SNX27-retromer cargoes to the plasma membrane by blocking their transport to lysosomes and to the TGN [91]. SNX27 also shares several structural similarities with SNX17: for instance, both proteins can bind cargoes with NPxY or NxxY motifs. SNX17 has been shown to be part of a recently-identified retriever complex. This is a novel, evolutionarily-conserved protein complex that is essentially a heterotrimer composed of DSCR3, C16orf62 and VPS29, and which associates with the CCC complex, the WASH complex, SNX17 and SNX31, assisting with the retrieval and recycling of NPxY/NxxY-motif-containing cargo proteins [92].

Retromer is considered to be critical for retrograde trafficking of the HPV-16 viral cargo to the TGN [58,72]. HPV-16 L2 interacts with the retromer through its retromer-binding sites (FYL at position 446–448 and YYML at position 452–455). Mutation of these sites abrogates retromer binding to L2, which, in turn, allows the accumulation of L2/vDNA in early endosomes and blocks the trafficking of viral cargo to the TGN [72]. SNX27 has also been shown to be involved in L2/vDNA trafficking. HPV-16 L2 (192–292 aa) binds to the PDZ domain rather than to the NPxY or NxxY motif of SNX27 [93], whereas, in the case of SNX17, the L2 NPxY region (position 254–257) has been shown to bind to the FERM domain of SNX17. This interaction assists the entry of the viral genome into the nucleus [44], possibly by retaining L2/vDNA in the endosome, and as a consequence this prevents the degradation of the cargo in the lysosomal compartment [44]. Knockdown of SNX17, or mutation of the NPxY motif, decreases viral infectivity as well as abrogating L2/vDNA trafficking. However, knockdown of SNX27 only causes a marginal reduction in infection, but, interestingly, knockdown of both SNX17 and SNX27 results in significant reduction in infection, greater than the effect observed for SNX17 knockdown alone. The L2 region that binds SNX27 is different from the region that binds the retromer complex, suggesting that L2-retromer binding is not mediated solely through SNX27, and possibly explaining why the depletion of SNX27 is insufficient to inhibit retromer-dependent retrograde trafficking. In addition, depletion of the DSCR3 and C16orf62 components of the retriever complex, as well as the CCC components CCDC 22 and CCDC93, results in a significant reduction in HPV infection [92]. This clearly indicates the role of the retriever complex in establishing HPV infection. As discussed earlier, the retromer complex has been shown to associate with SNX27 [90], whereas the retriever complex associates with SNX17. This raises the possibility that cooperative interaction of L2 with SNX17 and SNX27 might promote retromer-dependent TGN transport and is supported by a previous study showing SNX17 promoting retromer-dependent recycling of Jag1 [94].

In summary, the L2 protein interacts with a number of the above-mentioned cytosolic cellular sorting factors to route the viral cargo towards the TGN. As mentioned above, a critical aspect to this activity of L2 is its ability to span the endocytic membrane. How L2 does this is not well known but it is essential for the whole infectious process. One early study identified a conserved membrane-destabilizing peptide near the C-terminus of L2 (position 451–464 for HPV-16), which has a role in the endosomal escape of L2/vDNA via membrane disruption or destabilization [95]. Intriguingly, a recent study has shown that the basic segment that is present in the membrane-destabilizing peptide of L2 corresponds to a cationic cell-penetrating peptide (CPPs) [96]. This is highly conserved among diverse PVs and drives the part of L2 through the endosomal membrane into the cytoplasm [96], thus providing a mechanism by which L2 can access the cytosol and recruit cellular sorting factors. A transmembrane-like domain (TMD) closer to the N-terminus of L2 (at position 45–67 of HPV-16) has also been identified [97], and it is suggested that residues upstream of this TMD are lumenal, whereas the majority of the L2 is cytosolic thereby conferring interaction with host cell factors [98]. All of this is consistent with a type-I transmembrane topology and is in agreement with the SNX17, retromer and chromatin binding regions of L2. However, it remains to be determined whether L2 cleavage by cellular furin triggers conformational changes that enable L2 to insert and protrude into membrane using the TMD [99]. A translocation study using the PsV encapsidating L2-BirA suggests a different model for L2 topology [100] during HPV infection, where incoming L2/vDNA is trapped at the TGN in the presence of the S-phase inhibitor aphidicolin. This is a reversible effect, as removal of the inhibitor allows the cargos to egress from TGN in mitosis [100]. The inability of L2-BirA to translocate in the presence of inhibitor, suggests that the C-terminus is not cytosolic, which is not consistent with a with type I transmembrane topology. However, it implies that either L2-BirA is lumenal, or it becomes accessible to the cytosol once the cells enters mitosis. Based on this, it was suggested that L2-BirA might have a double-pass topology, where both N and C termini are lumenal, but most of the L2 protein is cytosolic [99]. However, L2 has only one TMD and no other membrane-spanning region has been identified so far, which makes this double-pass topology hard to conceptualize. Furthermore, this topology is in conflict with retromer binding. It is worth noting that currently there is no information on how many molecules of L2 are involved in trafficking of incoming viral genome and whether L2 might exist as a multimer. This obviously has major implications for how this membrane spanning activity of L2 might function. Clearly, much more work is needed to elucidate the conformation of L2 during virus infection and stoichiometry.

## 9. Post-TGN Transport

Once the L2/vDNA complex reaches the TGN [61], it is believed to remain there and wait to enter the nucleus. Cell cycle progression and mitosis are key events regulating this, step, with nuclear envelope breakdown being considered critical for L2/vDNA translocation from cytosol to nucleus [20,21]. After the onset of mitosis, the Golgi and TGN undergo fragmentation and vesiculation, and L2/vDNA-positive vesicles then egress from the TGN and associate with microtubules [101]. They then migrate along the microtubules towards the condensed chromosomes. It has been suggested that the L2 protein might interact with condensed chromosomes using its chromatin-binding domain [102] to ensure that the L2/vDNA vesicles remain at the mitotic spindle. In this model, L2/vDNA-positive mitotic vesicles would remain bound to chromosome for an extended period until the L2/vDNA translocates in the G1 phase after completion of mitosis. This model of L2/vDNA trafficking on microtubules suggests that L2 might be in the protruding conformation during this time, as that would allow the L2 spanning across the membrane to walk the viral cargo along the mitotic spindle [22]. The Campos laboratory has used the L2-BirA system to show that L2 translocation requires TGN localization of L2/vDNA and that it happens before the completion of mitosis [100]. While further work is needed to validate the exact mechanism by which L2/vDNA exits from TGN to nucleus, the cargo is eventually found to be localized to punctate nuclear foci called promyelocytic leukemia (PML) bodies, or ND10 (nuclear domain 10), in the interphase of the infected cell [23], where initiation of viral gene expression is believed to occur. L2 has a SUMO interacting motif and this is believed to play a role in PML localization [103]. It is also thought that localization to PML may contribute towards protecting the viral genome, [104], although some PML components such as Sp100 have been shown to display antiviral activity [105]. Interestingly a recent study also identified Myb-related transcription factor, partner of profilin (MYPOP) as an interacting partner of L2, and this was found to be a restriction factor for HPV infection [106]. Taken together, this indicates a very complex interplay between the virus and the host cells in the initial stage of viral infection and the onset of viral gene expression and replication.

## 10. Do Capsid Proteins Have a Role in Endocytic Transport in Late Infection?

During the PV life cycle, the expression of L1 and L2 proteins plays a role at two points: firstly, during virus entry L1 and L2 are essential to establish infection, as discussed in the above sections of this review; and later, at the end of the virus life cycle, where the capsid proteins are expressed in cytoplasm and translocated to the nucleus for capsid assembly and the encapsidation of the newly amplified viral genome. How the L1 and L2 assemble within the nucleus is not well understood. The interaction of cellular nucleophosmin with L2 protein is important for the correct assembly of infectious capsids [107]. The genome packaging involves the recruitment of L2 protein, facilitated by the viral E2 protein, to the ND10, prior to L1 recruitment [108]. However, the selectivity of viral encapsidation is still not fully understood, since L1 + L2 pseudovirions can package any nucleic acid 8 Kb in size, whereas in natural virions only the viral genome is retained, suggesting an, as yet, unknown selection mechanism for genome packaging. Daxx proteins might have a role in the recruitment of L2 to ND10 [109,110], whereas the L1 protein is later translocated to ND10 by the L2 protein [111]. Heat shock cognate protein 70 (Hsc70) has also been shown to facilitate the nuclear translocation of L2, and once virus assembly has taken place, Hsc70 displaces from virions [112]. Considering the roles played by L2 and L1 in virus entry and establishment of infection by taking advantage of cellular sorting machinery, it is possible that the capsid proteins might also be using some of the elements of endocytic trafficking at later stages of the virus life cycle to support either virus assembly, encapsidation or release. For example, L2 is able to change the localization of the cytoskeletal adaptor protein OBSL1 [41]. This may affect endocytosis or other trafficking and membrane dynamics. Recruitment of Hsc70 by L2 into the nucleus in the upper layers of the epithelium might also affect endocytic transport, as Hsc70 is involved in multiple different endocytic processes [113,114]. Moreover, SNX17 is a major cytosolic interacting partner of L2 [44] and since L2 is able to shuttle between the cytoplasm and the nucleus due to the presence of NLS and NES [115,116] it is possible this could modulate SNX17 normal function during the later stages of viral infection.

## 11. The Papillomavirus Oncoproteins and Endocytic Trafficking

While a reasonable amount is known about the role of endocytosis in HPV entry, very little is known about how viral oncoproteins might affect endocytosis during the HPV life cycle, or what contribution this might make to the development of malignancy.

Epithelial cell carcinogenesis involves many steps, such as the loss of cell polarity, shifts in presentation of polarized proteins, dynamic changes in cell morphology, increased proliferation and increased cell motility and invasion. All of these processes can be controlled by endocytic trafficking. Although mutations in the proteins involved in trafficking may not be direct drivers of transformation, imbalance in dynamic vesicle trafficking processes may have important roles, both in the initiation of transformation and the process of tumor cell invasion [117]. In this part of the review, we discuss some of the mechanisms through which papillomavirus oncogenic proteins can modulate endocytic transport pathways, potentially leading towards cancer development.

## 12. The Role of Viral Oncoproteins in HPV-Induced Carcinogenesis

The oncogenic potential of any HPV type is dependent on the three viral early proteins: E5, E6 and E7. The major oncogenic properties of high-risk, mucosotropic HPVs are provided by the E6 and E7 proteins. However, their oncogenic activity is enhanced by E5, which also plays an important role in tumor progression [118]. E5 increases the immortalization potential of HPV-16 E6 and E7 [119], and cooperates with HPV-16 E7 to stimulate the proliferation of human and mouse primary cells [120,121].

Unlike E6 and E7, much less is known about E5’s mechanism of action during transformation. Whilst E6 and E7 are the major oncoproteins of high-risk human papillomaviruses, E5 appears to be the major transforming protein of bovine papillomavirus (BPV) [122]. HPV E5 protein shows little homology with BPV E5, and has a much weaker transforming activity when it is expressed alone. Moreover, HPV E5 is not directly essential for the maintenance of the oncogenic phenotype, as it is not expressed in many HPV-positive tumors [122,123,124]. However, E5 does appear to be capable of contributing towards the early stages of tumor development, based on a variety of studies in tissue culture and transgenic models. Whether this is related to the ability of E5 to modulate growth factor receptor or immune avoidance remains to be determined. Tissue culture assays showed that HPV E5 can enhance the transforming activity of E6 and E7, suggesting that it may have a supportive role in tumor progression [119,120,121,125]. It has also been observed in transgenic mouse models that high-level expression of HPV-16 E5 in the skin induces epithelial hyperproliferation that results in spontaneous tumor formation. Interestingly, in estrogen-treated mice, the expression of E5 alone can induce cervical cancer [118,125]. All these observations suggest that E5 can act as an oncogene.

While the function of E5 in HPV-induced malignancy is still enigmatic, it is well known that E6 and E7 contribute directly towards the development of malignancy. The coordinate and continued expression of E6 and E7 is required for the maintenance of the transformed phenotype. Studies in tumor-derived cells showed that the loss of expression of either E6 or E7 induces cell growth arrest and apoptosis [126]. These two factors act cooperatively in the development of HPV-linked cancers, with the action of one factor complementing that of the other. E7 reprograms the infected cell to enter S-phase by targeting, in part, the pRb family members, thus allowing the E2F family of transcription factors to activate the transcription of various cell cycle progression genes [1,127,128]. The E6 oncoprotein complements the activity of E7 by suppressing the cell’s pro-apoptotic response to unscheduled DNA replication and targets pro-apoptotic proteins such as p53 [129] and Bak [130] for proteasome-mediated degradation, via the action of the E6AP ubiquitin ligase [131].

## 13. E5: Manipulation of Trafficking Pathways and Cancer Development

E5 is expressed by most HPV types except those of the β, µ and γ groups [124,132]. High-risk HPV types linked with malignant carcinomas, such as HPV-16, HPV-18, and HPV-31, possess a conserved E5 protein of around 80 amino acids (E5 alpha), whereas the low-risk HPV types associated with benign lesions (e.g., HPV-6 and HPV-11) contain two putative E5 proteins (E5 gamma and E5 delta), which have little sequence conservation [133].

E5 is a small, three-pass transmembrane protein, which is highly hydrophobic and has a cytoplasmic C-terminus [134]. It localizes primarily to the endoplasmatic reticulum (ER), but it can also be found in the Golgi apparatus (GA), in perinuclear regions and on the plasma membrane [135]. The cellular localization pattern of E5 suggests that its activity may be related to the trafficking of cytoplasmic membrane proteins through this cellular compartment. Recently, E5 has also been classified as a viroporin- a channel-forming viral membrane protein, able to modulate ion homeostasis and to play a critical role in many processes, including vesicle trafficking and viral life cycle [136,137].

**Figure 2:**
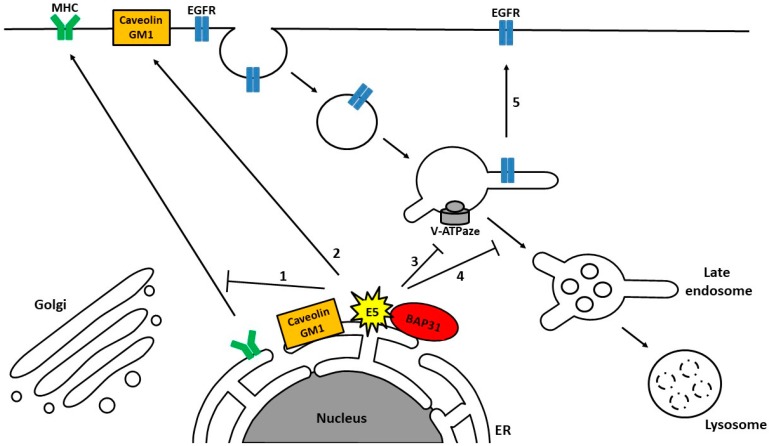
Model representing possible molecular mechanisms by which E5 manipulates trafficking pathways and potentially predisposes the infected cell towards cancer development. E5 inhibits the transport of immune receptors (MHCI, MHCII, CD1d) to the cell surface, and prevents clearance of infected cells by the immune response (1). It also upregulates the cell surface expression of caveolin-1 and ganglioside GM1 (2). Moreover, E5 can inhibit endosome acidification (3) or the trafficking from early to late endosomes (4) which may promote recycling of EGFR to the cell surface and lead to aberrant proliferation (5). Note that E5 localizes primarily to the endoplasmatic reticulum (ER), but it can also be found in the Golgi apparatus, in perinuclear regions and on the plasma membrane.

The exact mechanism of E5 activity in cellular transformation is not well understood. While BPV E5 mediates its carcinogenic effects via an association with the Platelet-derived Growth Factor Receptor (PDGFR), HPV E5 does not interact with the PDGFR [124,132]. However, some of the primary targets for HPV E5 are the members of the epidermal growth factor receptor family (EGFRs), which have been characterized as mediators of a wide variety of signal transduction events that control cell proliferation, migration, differentiation and survival. Inappropriate activation of the EGFR is one of the well-known signaling pathways involved in cancer progression [138,139]. Indeed a number of in vitro studies have demonstrated that the contribution of E5 toward host cell transformation appears to be dependent on manipulation of growth factor receptors and their downstream signaling pathways [121,140,141,142]. The precise mechanism by which E5 manipulates the EGFR is unclear. The first hypothesis was based on the interactions identified between E5 and the 16 KDa subunit of the vacuolar H+-ATPase (v-ATPase), which are conserved between different papillomaviruses [135]. Initially it was hypothesized that binding of HPV-16 E5 to 16 K impairs V-ATPase function, which leads to decreased acidification of the endosomes followed by increased recycling of the EGFR to the cell surface and a subsequent heightened receptor activation in response to EGF [142,143,144]. However, other studies suggest that HPV-16 E5 influences EGFR transport by altering trafficking from early to late endosomes through a mechanism that is pH-independent and might require reorganization of the actin cytoskeleton (Figure 2) [145,146].

E5 can also modulate other receptors such as keratinocyte growth factor receptors/fibroblast growth factor receptors 2b (KGFR/FGFR2b), which are required for the regulation of cell differentiation. In addition to the activation of EGFR, HPV-16 E5 down-regulates KGFR levels and its downstream signaling pathways. Since KGFR/FGFR2b is expressed primarily in the suprabasal cells of the epithelium, the effects of 16E5 on this receptor might perturb keratinocyte differentiation. However the mechanism by which E5 achieves this modulation is not understood [147,148].

It has been reported that E5 plays an important role in modulating trafficking of the major histocompatibility complex (MHCI), an activity which appears to be highly conserved between different papillomaviruses. The way that E5 prevents the transport of immune receptor MHC class I molecules to the cell surface—by retaining them in the Golgi apparatus and ER—also suggests that E5 has a function in intracellular trafficking (Figure 2) [149], and various mechanisms regulating this process have been suggested [150]. The direct interaction between the first transmembrane domain of E5 and the heavy chain component of MHC class I (HLA-I) could lead to a block in the trafficking of MHC class I molecules to the cell surface [151,152]. Other studies have proposed the formation of a ternary complex consisting of E5, MHC class I heavy chain and the chaperone calnexin, thereby allowing E5 to downregulate surface MHC class I expression [153]. Additionally, the observed interaction between E5, MHC class I, and chaperone B-cell receptor-associated protein 31 (BAP-31), which is a regulator of membrane protein transport, with its binding partner A4, may also play a role in this process. [154,155]. It has also been shown that E5 can target other immunologically relevant cell surface proteins, including MHC class II [156], and CD1d [157]. It has been suggested that HPV-16 E5 can perturb MHC class II maturation by inhibiting endosomal processing of the invariant chain component of MHCII, thereby blocking peptide loading and the subsequent transport of MHCII to the cell surface [156]. The role of E5 in immunological receptor trafficking is likely to contribute towards HPV immune evasion and establishment of a persistent infection. Indeed, HPV-16 E5-expressing cells are poorly recognized by CD8+ T cells, implying that down-regulation of HLA-I by 16 E5 inhibits immunopresentation of viral peptides and decreases the adaptive immune response to virally infected cells [158].

In addition, it has been observed that the expression of HPV-16 E5 changes the lipid composition of cellular membranes and increases the cell surface expression of caveolin-1 and ganglioside GM1 (Figure 2). This action of E5 might also affect cellular trafficking and cellular signaling, contributing to cancer development [159,160]. Indeed, it has been shown that higher levels of GM1 on the cell surface can augment the proliferative response to EGF [161].

Despite the postulated role of E5 in modulation of trafficking pathways, the exact mechanism of action of E5 remains to be elucidated. Its subcellular localization, either at the ER or the Golgi, and the fact that it is a transmembrane protein with a cytoplasmic C-terminus suggest that it might be able to target other proteins that are involved in the regulation of endocytic transport. Interestingly recent proteomic studies have indicated that E5 could potentially interact with proteins which might regulate many steps of vesicular trafficking, such as Rab GTPase (Rab18, Rab32, Rab34), Sorting nexin family proteins (SNX4, SNX14, SNX19), coatomer subunits (COPA, COPB, COPE), vesicle associated membrane protein (VAPA and VAPB), ERGIC1, Golt1b, SURF4, PRAF2, ZFPL1 and RER1 [162].

Many of E5’s interacting partners—ERGIC1, Golt1b, SURF4, PRAF2, and ZFPL1—are involved in regulating ER-to-Golgi transport, whereas others such as the coatomer subunits and RER1 protein, control retrograde transport from the Golgi to the ER [163,164,165,166,167,168].

Rab GTPases are components of the vesicle trafficking machinery. They control vesicle formation, movement along actin and tubulin networks and also docking and membrane fusion events [169,170]. Taken together, it would appear that E5 is a prime candidate for modulating endocytic transport pathways through variety of different mechanisms.

## 14. Novel and Unexpected Role of E6 in the Regulation of Endocytic Transport

The E6 oncoprotein is, approximately 18 kDa in size and is generally thought to be localized in the nucleus, but it can also be found in cytoplasm [171]. Interestingly, it has been reported that the bulk of endogenously expressed HPV18 E6 is found in the membrane fraction of HeLa cells, with smaller amounts present within the cytosolic and nuclear compartments [172,173,174]. A unique characteristic of the cancer-causing E6 oncoproteins is the presence of a PDZ binding motif (PBM) at the carboxy termini, which is absent from benign HPV types, indicating that the PBM is a signature for oncogenic potential [175]. An intact E6 PBM is important for the ability of E6 to cooperate with E7 in the generation of tumors in transgenic mouse models, and also has transforming potential in different tissue culture models [176,177,178]. In the context of the whole viral genome, loss of E6 PBM function results in a defective replicative life cycle, with reduced levels of viral DNA amplification and, ultimately, loss of the viral episomes [179,180].

Several studies have addressed how the interaction between E6 and its cellular PDZ targets might contribute towards the development of malignancy [181,182,183]. Many cellular PDZ domain-containing targets of E6 have been reported. One of the best-characterized E6 PBM interacting partners is a group of proteins involved in the regulation of cell polarity and potential tumor suppressor proteins such as Discs Large (DLG1) and Scribble (hScrib) [184,185,186]. However, recent studies have also shown a novel and unexpected role of E6 in the regulation of endocytic trafficking [187].

The endocytic machinery plays important roles in epithelial organization and polarity control [188,189]. Although the direct effects of E6 on cell polarity proteins such as Dlg1 and Scribble have been studied, the interplay among E6, its targets and trafficking is still not well understood.

Recent proteomic analyses have suggested that HPV-18 E6 could potentially interact with several different components of the endocytic sorting machinery, including the retromer components (Vps26, Vps29 and Vps35) and SNX27, indicating that E6 might play an important role in the regulation of cellular trafficking pathways [162,190]. Indeed, SNX27 has been identified as another PDZ domain-containing target of E6. As mentioned before, SNX27 interacts with the retromer complex and controls the correct trafficking of a large number of PBM-containing cargoes, such as transmembrane receptors, transporters and ion channels [90,162,190]. Some of the well-known SNX27 targets include the Na/H exchanger regulatory factor (NHERF) [191], the Cytohesin-associated scaffolding protein (CASP) [192], the junctional protein zonula occludens-2 (ZO-2) [193], and the β-2 adrenergic receptor (β2AR) [194,195,196]. Many have been shown to be recycled by E6 in a PBM-dependent manner. In support of this, SNX27 has been found to interact with several different high-risk HPV E6 proteins in a PBM-dependent way. However, ablation of the E6 PBM or the core region of the SNX27-PDZ reduced their interaction significantly, but not completely, suggesting that an additional motif is important for their association. In support of this, a weak but consistent interaction was also observed between the low risk HPV-11 E6 protein and SNX27 [187].

It has been shown that, unlike most of its PDZ domain-containing targets, E6 does not target SNX27 for degradation. It has been demonstrated rather, that in HPV-18 positive cell lines the association of SNX27 with components of the retromer complex and the endocytic transport machinery is altered in an E6 PBM-dependent manner. Analysis of a SNX27 cargo, the glucose transporter GLUT1, reveals an E6-dependent maintenance of GLUT1 expression, and alteration in its association with components of the endocytic transport machinery. Furthermore, knockdown of E6 in HPV-18-positive cervical cancer cells phenocopies the loss of SNX27, both in terms of GLUT1 expression levels and its vesicular localization, with a concomitant marked reduction in glucose uptake, whilst loss of SNX27 results in slower cell proliferation in low nutrient conditions.

This suggests that E6 interaction with SNX27 can alter the recycling of cargo molecules, one consequence of which is modulation of nutrient availability in HPV-transformed tumor cells. All these observations indicate a new and unexpected activity of the E6 oncoprotein, linking it directly to the regulation of endocytic transport pathways.

**Figure 3:**
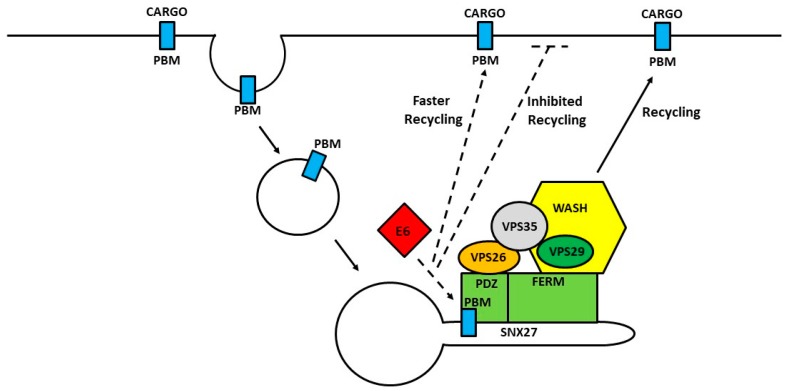
Working model showing the possible mechanism of modulation of SNX27-retromer mediated cargo recycling by HPV E6. After internalization, cargoes containing a PDZ-binding motif (PBM) bind to a PDZ domain of SNX27. SNX27 serves as an adaptor linking its cargoes to the endosomal tubules through its interaction. Association within the SNX27-retromer-WASH complex directs the cargoes towards recycling to the plasma membrane. The E6-SNX27 interaction may be a transient step in the handover in the SNX27-retromer complex for cargo binding, which may in turn promote faster recycling or inhibit recycling of certain PBM-containing cargoes. It is still unknown if E6 helps recruit SNX27 to certain endocytic compartments or competes for binding with SNX27 cargoes. Solid line shows the role of the SNX27-retromer complex in cargo recycling. The dashed line shows how E6 might affect this.

Several major questions now arise from these studies. What is the precise mechanism by which E6 can modulate SNX27 function? The SNX27-PBM interaction seems to be PDZ-PBM mediated, but not exclusively. Does the E6 PBM help recruit SNX27 to certain endocytic compartments, favoring faster recycling? Does E6 compete for binding with SNX27 cargoes, depending on the function and requirement of these cargoes in the context of HPV life cycle (Figure 3)?

It is interesting that the bulk of E6 has been observed to be localized within the membrane compartment, but only a small portion is regulated by SNX27. This could suggest that other components of the endosomal system could be involved in E6 recruitment to the membranes. Further studies are needed to better understand this novel function of E6 in the modulation of cellular trafficking pathways.

## 15. Is There Any Link between E7 and the Endocytic Machinery?

E7, similar to E6, is a multifunctional protein which has been reported to interact with many cellular substrates, and, in many cases, to perturb their normal cellular function. The evidence of shuttling between nuclear and cytoplasmic compartments by E7 [173,197,198] correlates with its ability to interact with both nuclear and cytoplasmic binding partners [199,200]. However, whether E7 has any role in the regulation of endocytic pathways still needs to be elucidated. There is substantial evidence showing that deregulated endocytosis is a feature of many cancer types, and that the induction of many cancer hallmarks, such as the loss of cell polarity, cell-cell adhesion, deregulated metabolism, promotion of inflammation, increased proliferation, metastasis and invasion, and possibly others, is influenced by the perturbation of endocytic recycling machinery [117,201,202,203]. Many of these pathways, such as alteration of cellular energetics, modulation of cell death, avoidance of immune destruction, or activation of invasion or metastasis are also affected by E7 [204,205]. However, the crosstalk between E7 function and endocytosis is still an unexplored area of research. Recent proteomics studies showed that E7 interacts with some components of the endocytic machinery, but the function of these interactions is still unknown [162]. One of the interacting partners of E7 which could be involved in trafficking is the coatomer complex (COPA and COPE). This cytosolic protein complex is required for budding from Golgi membranes, and is essential for the retrograde Golgi-to-ER transport of dilysine-tagged proteins [206]. E7 was also found to interact with components of the adaptor protein complex 2 (AP-2), such as AP2A1, AP2A2, AP2B1, AP2S1, and AP2M1. The adaptor protein machinery is involved in protein transport via vesicles in different membrane traffic pathways.

Taken together, this suggests that E7 might also play important roles in trafficking regulation.

## 16. Conclusion and Future Directions

As discussed above, many host proteins and pathways are exploited by HPV to facilitate virion trafficking to establish virus infection. More work is required to clarify the remarkable roles of L1 and L2, both during the early and late infectious events. The structural studies of L2 will help in understanding the exact mechanisms involved in the escape of L2/vDNA from the endosome, their entry into the TGN and their translocation from the TGN to the nucleus. Moreover, studies should also focus on understanding how the HPV oncoproteins perturb endocytic transport during the development of cervical cancer. It is crucial to define the interaction between viral oncoproteins and endocytic components, to explain the mechanisms by which papillomavirus oncoproteins modulate endocytic cargo trafficking, and to understand the biological consequences of these activities. This will be very helpful to determine whether targeting these pathways has any potential for therapeutic intervention in HPV-induced malignancy, and may also define whether any of the identified cargoes might be prognostic markers of disease development.
